# Supplementary material for: The causal role of circulating inflammatory markers in osteoporosis: a bidirectional Mendelian randomized study
Source: Front Immunol. 2024 Jul 18;15:1412298. doi: 10.3389/fimmu.2024.1412298 (PMC11291241; doi:10.3389/fimmu.2024.1412298)
Supplement: Supplementary file 1 [file DataSheet_1.zip › supplementary material/OSTEOPOROSIS-CIM/GCST90274759.tsv.gz_leave_one_out.pdf]

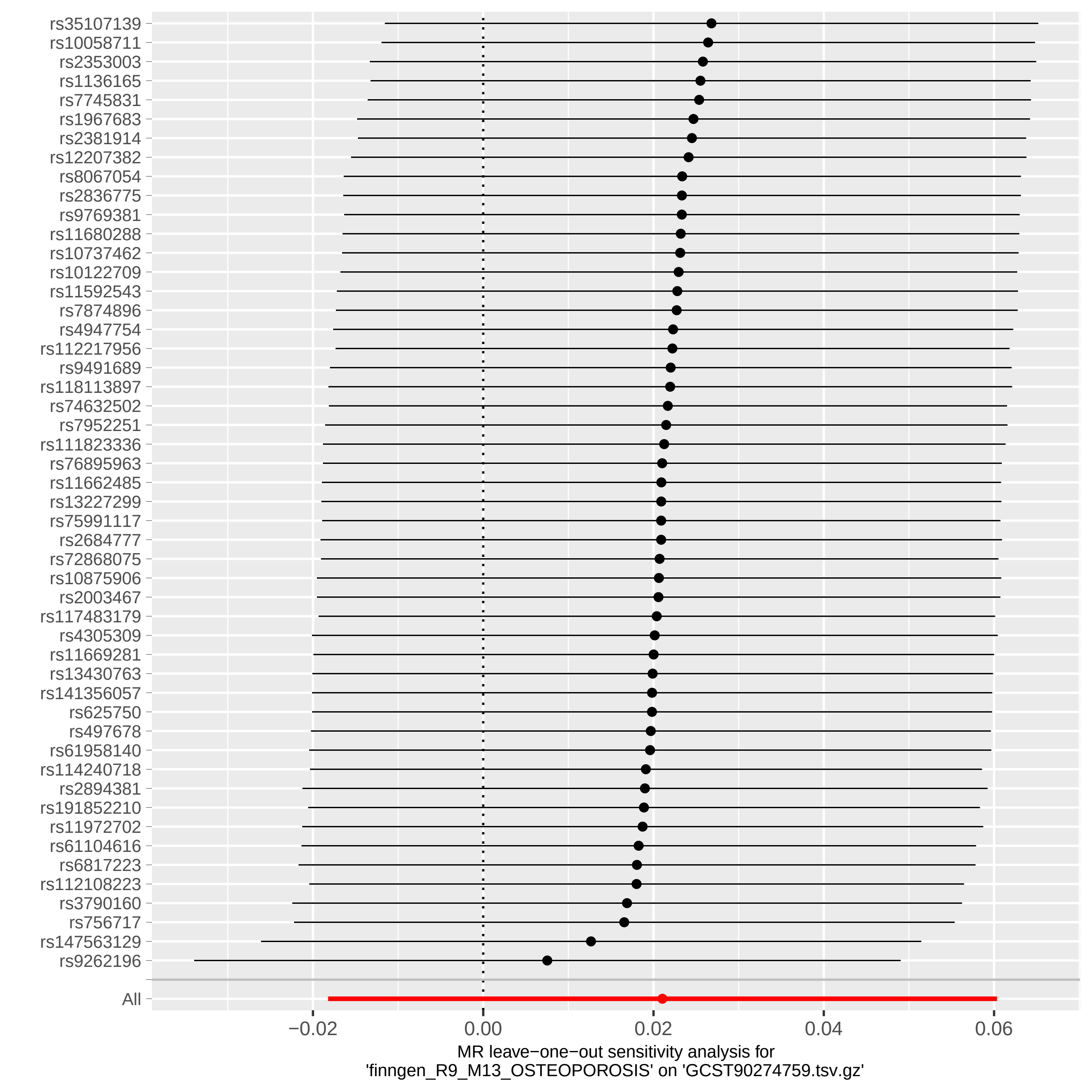
rs35107139
rs10058711
rs2353003
rs1136165
rs7745831
rs1967683
rs2381914
rs12207382
rs8067054
rs2836775
rs9769381
rs11680288
rs10737462
rs10122709
rs11592543
rs7874896
rs4947754
rs112217956
rs9491689
rs118113897
rs74632502
rs7952251
rs111823336
rs76895963
rs11662485
rs13227299
rs75991117
rs2684777
rs72868075
rs10875906
rs2003467
rs117483179
rs4305309
rs11669281
rs13430763
rs141356057
rs625750
rs497678
rs61958140
rs114240718
rs2894381
rs191852210
rs11972702
rs61104616
rs6817223
rs112108223
rs3790160
rs756717
rs147563129
rs9262196
All
−0.02
0.00
0.02
0.04
0.06
MR leave−one−out sensitivity analysis for
'finngen_R9_M13_OSTEOPOROSIS' on 'GCST90274759.tsv.gz'
